# Bioengineering 3D Pancreatic Cancer Models with Fibrotic Stroma for In Vitro Cancer Modeling

**DOI:** 10.3390/mi16101140

**Published:** 2025-10-02

**Authors:** Xingrun Lan, Keke Chen, Xiaoyun Wei

**Affiliations:** 1 School of Automation, Hangzhou Dianzi University, Hangzhou 310018, China; 2 Institute of Medical Robotics, School of Biomedical Engineering, Shanghai Jiao Tong University, Shanghai 200240, China; 3 Key Laboratory of Medical Information and 3D Bioprinting of Zhejiang Province, Hangzhou Dianzi University, Hangzhou 310018, China

**Keywords:** 3D PDAC model, bioengineering, fibrotic stroma, cancer modeling

## Abstract

Pancreatic ductal adenocarcinoma (PDAC) remains highly lethal due to late diagnosis, high malignancy, and profound resistance to therapy. Traditional two-dimensional (2D) cell cultures fail to recapitulate the complex tumor microenvironment (TME), especially the fibrotic stroma, which is crucial for the progression of PDAC and drug response. In vitro three-dimensional (3D) models, which provide more physiologically relevant features such as tight cell–cell and cell-extracellular matrix (ECM) interactions, as well as 3D architecture, have been regarded as highly promising models in PDAC research. This review summarizes some representative in vitro PDAC models, including 3D spheroids, tumor-on-a-chip, bioprinted constructs, and patient-derived organoids (PDOs), particularly focused on the advances in bioengineering strategies for the integration of the key stomal components for microenvironment recapitulation and their applications. Additionally, we discuss the current challenges facing 3D models and propose potential strategies for constructing in vitro models that more accurately simulate the pathophysiology of the fibrotic stroma, aiming for their application in clinical settings.

## 1. Introduction

Pancreatic cancer is a highly aggressive and lethal malignancy, with a 5-year survival rate of less 10% [1,2,3]. Its incidence and mortality rates are steadily increasing worldwide, and, by 2030, pancreatic cancer is projected to become the second leading cause of cancer-related deaths [4]. Among its subtypes, pancreatic ductal adenocarcinoma (PDAC) is the most common and poses the greatest clinical challenges. The lack of specific symptoms in the early stages of PDAC hampers timely diagnosis, resulting in most patients being diagnosed at an advanced stage. Currently, conventional chemotherapy remains the standard treatment for late-stage PDAC, offering only a limited extension of survival by a few months [5].

Research indicates that the unique biological structure of PDAC and its stromal components within the tumor microenvironment (TME) closely promote tumor progression and treatment [6,7]. During PDAC development, pancreatic cancer cells activate stromal components, primarily pancreatic stellate cells (PSCs), which are located in the periductal and periacinar regions, generating a large number of cancer-associated fibroblasts (CAFs). Further, a desmoplastic reaction is easily triggered, characterized by excessive deposition of the extracellular matrix (ECM). Ultimately, malignant PDAC cells become embedded within a dense fibrotic barrier, forming a stratified core–shell structure where the tumor mass is encapsulated by stroma [8]. This distinctive tumor–stroma niche governs the complex crosstalk interactions between pancreatic cancer cells and CAFs, further promoting cancer cell proliferation, migration, invasion, and drug resistance. Therefore, constructing 3D PDAC models to recreate the critical spatial characteristics of the tumor-stroma components is of great importance for pancreatic cancer research and drug screening.

Currently, an increasing number of 3D in vitro PDAC models are being developed with the advancement of engineering technology. The advent of 3D models has addressed the limitations of traditional 2D monolayer cultures and costly, low-throughput animal models. Current 3D models include the following: (1) self-assembled spheroids [9,10], (2) tumor-on-a-chip systems [11,12], (3) 3D-bioprinted constructs [13,14], and (4) patient-derived organoids (PDOs) [15,16]. By employing 3D tumor modeling approaches, it is possible to precisely incorporate elements of the TME, thereby enabling the investigation of the tumor–stroma dynamics interactions, as well as the identification of therapeutic targets that disrupt their tumor-promoting functions.

In this review, we present and discuss the current 3D modeling strategies for PDAC. We first provide an overview of the cellular and acellular components of the PDAC tumor microenvironment and their respective biological roles. We then examine a range of in vitro tumor models developed using different engineering approaches, highlighting their ability to faithfully recapitulate the heterogeneity of the TME, cell–cell interactions, and therapeutic responses. Finally, we discuss the advantages and current limitations of each model, offering insight into their potential for future applications.

## 2. Fibrotic Stroma of PDAC Tumors

**Figure 1:**
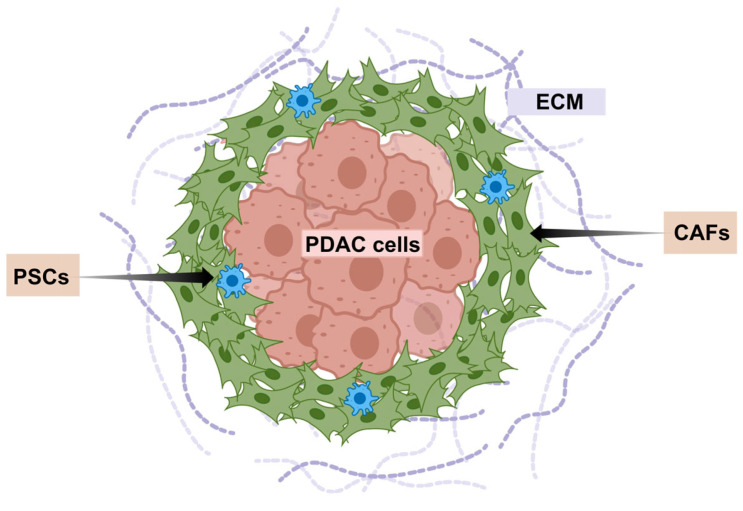
Schematic of characteristics of the PDAC stroma.

The exceptionally aggressive nature and profound therapeutic resistance of PDAC are inextricably linked to its unique and complex TME [17,18,19]. The PDAC TME constitutes a dynamic, highly interactive niche dominated by an extensive desmoplastic stroma (Figure 1). This dense fibrotic reaction, characterized by excessive deposition and remodeling of ECM components like collagen, fibronectin, and hyaluronic acid (HA), creates significant physical and biochemical barriers [20]. Crucially, this stroma is cellularly rich, heavily populated by activated CAFs, PSCs, vascular and immune cells [21]. The complex interactions between tumor and stroma contribute to tumor cell proliferation, invasion and metastasis.

### 2.1. ECM of PDAC

The ECM constitutes a fundamental and dynamic element of the PDAC TME. Beyond providing structural support, the PDAC ECM dynamically interacts with cellular components, critically regulating growth, behavior, and responses to stimuli [22]. The ECM in PDAC is primarily composed of type I collagen, followed by fibronectin and laminin. In addition, non-collagenous components such as glycoproteins, proteoglycans, and glycosaminoglycans also contribute to the ECM architecture [23]. During PDAC progression, a dense stromal matrix accumulates, accompanied by tissue stiffening. In advanced stages of pancreatic cancer, the fibrotic ECM can constitute up to 90% of the tumor mass [24]. This excessive stroma on the one hand supports tumor compartmentalization while simultaneously impeding drug perfusion and delivery. On the other hand, the dense, abnormal ECM architecture directly contributes to intratumoral hypoxia [25]. Moreover, pro-survival growth factors and cytokines embedded within the ECM further reinforce PDAC’s resistance to therapy [26].

Collagen is the most abundant and specific component of the PDAC ECM. Type I collagen is the predominant structural component of the PDAC ECM, accounting for 80–90% of the fibrillar content in the desmoplastic stroma [27,28]. Its aberrant deposition and cross-linking drive the characteristic tissue stiffening observed in PDAC, elevating matrix rigidity from healthy pancreatic tissue to malignant lesions increase approximately 2.6-fold [29]. This densely packed collagen network forms a physical barrier that severely restricts vascular perfusion, exacerbates hypoxia, and impedes chemotherapeutic drug delivery. Currently, 3D cell culture models employing collagen gels are widely utilized to investigate interactions between cells and the matrix [30,31]. Researchers mimic the stromal matrix and basement membrane by embedding PDAC cells in hydrogels composed of varying ratios of type I collagen and Matrigel. PDAC cells cultured in Matrigel display an epithelial phenotype, whereas type I collagen induces epithelial-to-mesenchymal transition (EMT), promoting an invasive phenotype due to increased matrix stiffness and fiber density [32]. However, the maximum stiffness tested in these studies is around 1 kPa, which is substantially lower than the 4–8 kPa range characteristic of PDAC tissue [33].

In addition, PDAC is characterized by the accumulation of HA, a non-sulfated glycosaminoglycan secreted by both tumor cells and CAFs [34]. Over time, HA progressively accumulates within the tumor stroma, resulting in altered tissue stiffness in malignant lesions [35]. Low levels of HA influence cellular dissemination and are associated with impaired drug delivery, as well as increased tumor invasiveness and metastatic potential. Patients exhibiting high HA expression typically have poorer survival outcomes [36]. HA-mediated motility is facilitated through the expression of the receptor for hyaluronan-mediated motility (RHAMM) and its interaction with CD44, which collectively promote tumor cell migration and invasion. CD44, a well-established stem cell marker, facilitates tumor metastasis by inducing the loss of E-cadherin and accumulation of β-catenin [37,38]. Moreover, HA upregulates the transcription factor NANOG and other stemness regulators, leading to the activation of multidrug resistance protein 1 (MDR1) and conferring chemoresistance in CD44-positive cells [39].

### 2.2. Stromal Cell of PDAC

CAFs are activated fibroblasts present within almost all solid tumors [40,41,42]. Fibroblast activation is a physiological response to tissue injury, wound healing, inflammation, and tumorigenesis [43]. In PDAC, a significant portion of CAFs originates from PSCs, acquiring a myofibroblast-like phenotype that closely resembles conventional wound-healing responses [44]. CAFs may also derive from bone marrow-derived mesenchymal cells or resident fibroblasts [45]. CAFs contribute to the formation of a dense ECM by secreting collagen, HA, and fibronectin, thereby increasing tissue stiffness, promoting the EMT process, and enhancing tumor invasiveness. The study demonstrated that fibronectin secreted by CAFs activates the PI3K/Akt signaling pathway to inhibit tumor cell apoptosis and, in cooperation with collagen, induces matrix metalloproteinase (MMP) expression, contributing to increased resistance to gemcitabine treatment [46,47,48].

The cellular heterogeneity and plasticity of CAFs in PDAC have been shown to be crucial in regulating gene expression, tumor development, and disease progression. This diversity in cellular origin contributes to the broad phenotypic and functional heterogeneity observed among CAF subtypes. Öhlund et al. identified two distinct CAF subpopulations in pancreatic cancer, including inflammatory CAFs (iCAFs) and myofibroblastic CAFs (myCAFs) [44]. As reported, the myCAFs located in close proximity to tumor cells are characterized by high α-SMA expression, active TGF-β signaling, and low secretion of inflammatory mediators. They are generally associated with tumor-restraining effects. In contrast, iCAFs reside more distally from tumor cells, promote tumor progression, and exhibit low α-SMA expression while secreting high levels of IL-6, IL-11, and other pro-tumorigenic chemokines. A deeper understanding of CAF biology is essential for the development of improved therapeutic strategies.

In addition, studies have shown that CAFs also play a significant role in the formation of the immunosuppressive microenvironment of PDAC [49,50]. The fibrous matrix layer formed by collagen deposition induced by CAFs can prevent CD8^+^ T cells from infiltrating the tumor, thereby inhibiting CD8^+^ T cell-mediated anti-tumor immunity. At the same time, the cytokines, such as IL-6, IL-8, CXCL12, and CXCL8, secreted by CAFs, have also been proven to be important factors in suppressing the anti-tumor immune response of pancreatic cancer [51]. The immunosuppressive microenvironment results in the significantly lower-than-expected efficacy of conventional immunotherapy (such as immune checkpoint inhibitors) in PDAC [52]. Thus, the construction of 3D models that mimics the key stroma properties of the TME of the PDAC is of great significance for in-depth research on chemotherapy, immunotherapy, and combined treatment regimens for pancreatic cancer, as well as for exploring effective treatment approaches.

## 3. Bioengineering 3D PDAC Models

### 3.1. Three-Dimensional Spheroids

Three-dimensional spheroids represent a significant advancement over traditional 2D monolayer cultures for modeling PDAC [53,54]. The concept of spheroids is most commonly defined as 3D cellular aggregates formed through self-assembly of cancer cell lines and was first proposed by Sutherland and colleagues in the 1970s. Since then, various methods for spheroid generation have been developed [55]. Several distinct spheroid culture techniques currently exist, including suspension culture, liquid overlay, low-adhesion surfaces, hanging-drop methods, microwell, and microfluidic approaches [56,57,58]. Despite methodological differences, the underlying principle of spheroid formation remains consistent, that is, the intrinsic ability of cells to aggregate into 3D clusters driven by cell–cell contacts and ECM secretion [59]. Spheroids spontaneously develop key features absent in 2D, including pronounced cell–cell, cell–matrix interactions and enhanced deposition of the ECM. The dense architecture of 3D spheroids typically exhibits nutrient and oxygen gradients similar to those found in vivo, enhancing their biomimicry in modeling intrinsic PDAC characteristics such as profound chemoresistance.

**Figure 2:**
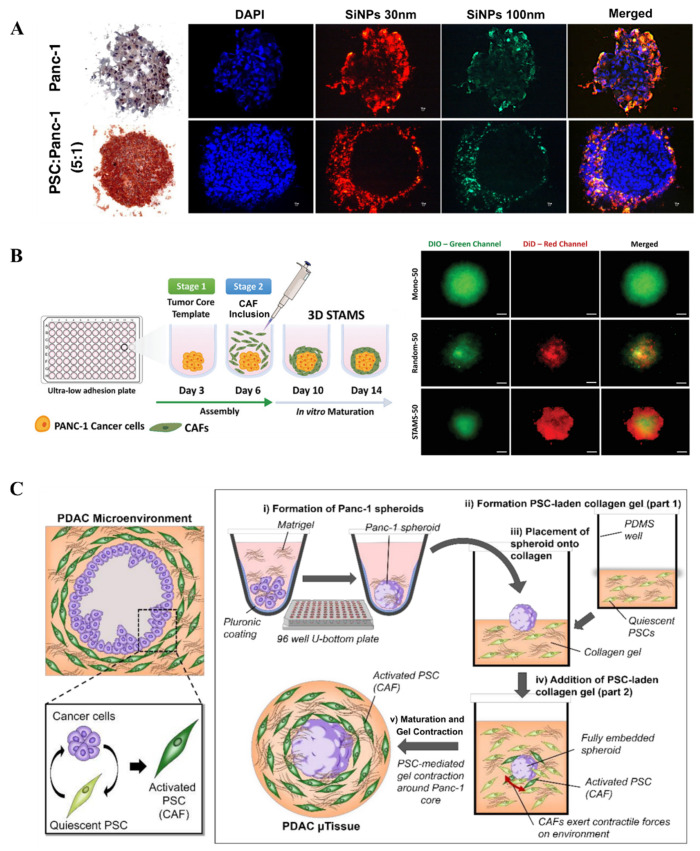
Fabrication of 3D PDAC spheroids: (**A**) Fluorescence images of 3D human pancreatic tumor models established using Panc-1 cell line alone and co-culturing Panc-1 cells and pancreatic stellate cells (PSC) with fluorescent silica nanoparticle (SiNP) penetration (reproduced from Reference [60]). (**B**) Schematics of 3D-stratified PDAC models assembly by using ultra-low adhesion plates with a cell ratio of PANC-1: CAF fixed at 1:4 (reproduced from Reference [61]). (**C**) Schematic representation of the cellular arrangement of pancreatic cancer cells and pancreatic stellate cells (reproduced from Reference [63]).

To better recapitulate critical aspects of the in vivo tumor microenvironment more faithfully, recent PDAC spheroids frequently incorporate stromal cells, such as CAFs or PSCs, to better represent tumor–stroma interactions. For example, Priwitaningrum et al. employed centrifugation to aggregate Panc-1 and PSCs within microwells, generating heterotypic spheroids (Figure 2A) [60]. After 48 h, spheroid formation was confirmed, and their application in drug screening was further evaluated by incubating with silica nanoparticles. Notably, nanoparticle penetration was significantly reduced in the presence of CAFs, demonstrating barrier functions analogous to those observed in vivo. To simulate the fibrotic stroma layer structure of PDAC, João F. Mano’s group fabricated stratified co-spheroids by utilizing ultra-low attachment plates (Figure 2B) [61]. They first allowed pancreatic cancer cells to self-assemble into 3D spheroids. Subsequently, CAFs were seeded onto the surface of these preformed spheroids. Using the liquid overlay technique, they ultimately established a semi-enclosed, matrix-rich outer layer surrounding the tumor core, which was then applied in chemotherapy drug testing studies. Compared to heterotypic spheroid models with a randomly mixed co-culture, the stratified spheroid model demonstrated significantly stronger drug resistance. This validates the role of the dense stromal cell layer in enhancing pancreatic cancer cells’ ability to resist drug treatment. For more complex microenvironment modeling, Lazzari et al. introduced endothelial cells (HUVECs) into PDAC heterotypic spheroids composed of Panc-1 tumor cells and MRC-5 fibroblasts to simulate the vascular components found in vivo [62]. Although the inclusion of HUVECs did not result in vascular network formation, these spheroids exhibited increased chemoresistance. This study marked the first attempt to integrate multiple stromal cell types within spheroid models. Kunal P. Pednekar et al. developed a novel 3D PDAC microtissue model by embedding Panc-1 tumor spheroids into a collagen hydrogel enriched with CAFs, effectively mimicking the dense fibrotic stroma that surrounds tumor cells in PDAC patients (Figure 2C) [63]. This model leverages the contractile properties of CAFs to create in vivo–like spatial architecture and exhibits a gene expression profile similar to that of PDAC patients. It provides an efficient and controllable platform for studying tumor–stroma interactions and for evaluating antifibrotic drugs such as the ITGA5 antagonist peptide AV3.

Despite their ease of generation and ability to incorporate one or more stromal components, spheroids have limitations. A primary challenge is the lack of comprehensive control over spheroid architecture. In heterotypic spheroids composed of tumor cells and fibroblasts, fibroblasts often aggregate more rapidly and densely, resulting in a spheroid morphology where tumor cells envelop a fibroblast-rich core [64,65,66]. This arrangement contrasts with the in vivo scenario, where epithelial tumor cells are surrounded by a fibroblast-rich stroma. Another limitation lies in the restricted diversity of cell types and the difficulty in generating sufficient ECM to support cell aggregation. For example, incorporating macrophages remains challenging due to their propensity to degrade rather than produce ECM [67]. Despite these constraints, spheroids remain among the simplest 3D in vitro models, offering a high-throughput platform for evaluating novel PDAC therapies and broad utility in research applications.

### 3.2. Tumor-on-a-Chip

Another emerging area in 3D tumor modeling is the use of organ-on-a-chip or microfluidic-based platforms. These systems typically feature intricate microstructures, such as interconnected chambers or channels, often with dimensions on the micrometer to millimeter scale, allowing cells to grow within spatially confined environments [68,69,70]. The concept of “organ-on-a-chip” was first introduced in a seminal 2007 publication and rapidly gained widespread recognition due to its immense potential in elucidating fundamental pathophysiological processes and therapeutic responses [71]. Notably, organ-on-a-chip platforms enable the incorporation of physiological fluid flow, shear stress/mechanical cues, as well as spatial and chemical gradients within the model. Their modularity and compatibility with optical and fluorescence microscopy make them highly adaptable, while microfluidic systems can be readily integrated with high-throughput screening and high-content imaging techniques, facilitating real-time monitoring of 3D tumor model maturation and drug response [72,73,74].

**Figure 3:**
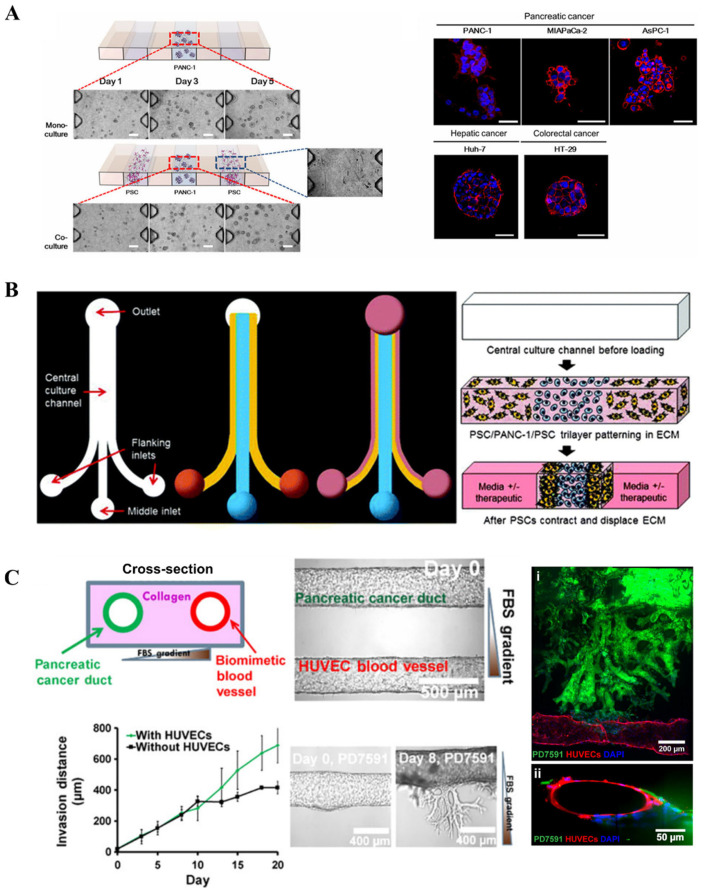
Tumor on chip: (**A**) Schematic of a tumor-on-a-chip organotypic model to study cancer cells’ vascular invasion (reproduced from Reference [75]). (**B**) Design and operation of the microfluidic device (reproduced from Reference [76]). (**C**) Schematic of spheroids in a collagen-supported microchannel plate when cultured (reproduced from Reference [77]).

More recently, Lee et al. produced a microfluidic chip-based 3D co-culture model to study the interactions between pancreatic cancer cells and PSCs within the tumor microenvironment (Figure 3A) [75]. The study found that co-culture with PSCs promoted tumor spheroid formation by Panc-1 cells, enhanced their migratory capacity, and induced the expression of EMT-related markers such as vimentin and TGF-β. Furthermore, the co-culture model recapitulated the chemoresistance of tumor cells to agents like gemcitabine, while combination treatment with paclitaxel and gemcitabine showed a synergistic inhibitory effect. Drifka et al. proposed a platform in which pancreatic cancer cells are cultured in a configuration surrounded by PSCs, encapsulated within a collagen-based hydrogel to establish a 3D structure (Figure 3B) [76]. This system also incorporates perfusable channels mimicking vasculature, enabling the administration of therapeutic agents. The model successfully demonstrated ECM deposition and cell–ECM interactions. Upon treatment with paclitaxel, a dose-dependent reduction in cell viability was observed, along with a notable decrease in ECM density within the model. A dual-channel microfluidic chip was developed featuring two parallel hollow cylindrical channels embedded within a collagen matrix for studying PDAC–vascular interactions (Figure 3C) [77]. One channel was seeded with pancreatic cancer cells, while the other contained endothelial cells, establishing distinct tumor and vascular compartments. During culture, pancreatic cancer cells were observed invading the endothelial channel matrix, originating from tumor cells and intraluminal endothelial cells, leading to endothelial ablation. This phenomenon was corroborated in vivo, confirming that PDAC tumors invade vasculature and ablate endothelial cells in both settings. Furthermore, the chip platform demonstrated that inhibition of transforming growth TGF-β receptor signaling reduced PDAC-mediated endothelial ablation. Given that tumor vascular remodeling is considered a potential therapeutic target, these findings offer novel insights and strategies for future 3D modeling of pancreatic cancer.

PDAC-on-chip models represent a rapidly evolving frontier. By integrating critical physiological parameters like flow, spatial organization, and multi-cellular complexity, they provide a more faithful in vitro representation of the human PDAC TME than previous models. Research utilizing these platforms, as exemplified above, is elucidating fundamental mechanisms of desmoplasia, metastasis, and therapy resistance driven by stromal interactions, vascular dysfunction, and immune evasion. This enhanced physiological relevance makes them powerful tools for target discovery, drug screening, and, ultimately, improving the translation of preclinical findings to clinical success.

### 3.3. Three-Dimensional Bioprinted PDAC Constructs

Three-dimensional bioprinting, a novel additive manufacturing technique, has gradually developed over the past decade and rapidly gained widespread application in the field of tissue engineering. It enables the precisely controlled assembly of cells, hydrogels, bioactive molecules, and other substances to form complex 3D tissue constructs, offering significant advantages for building tissue models [78,79]. According to the manufacturing principle, 3D bioprinting techniques can be regarded as inkjet bioprinting, extrusion bioprinting, laser-assisted bioprinting, and light-cured bioprinting [80]. The development of 3D bioprinting facilitates the generation of PDAC models with precise spatial control over TME components, through controllable patterning of cancer cells, stromal cells, and ECM-mimicking hydrogels to recapitulate the desmoplastic stroma characteristic of PDAC.

**Figure 4:**
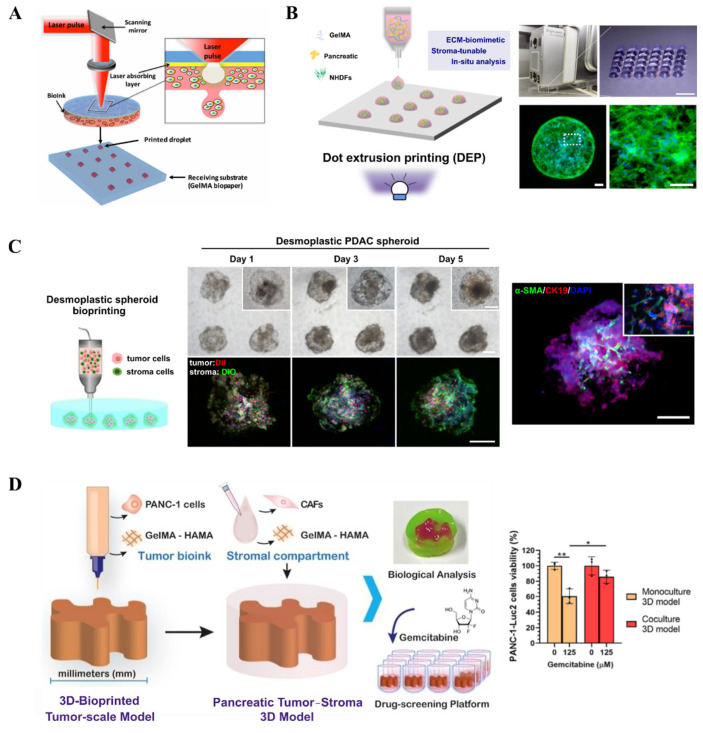
Bioprinting of 3D PDAC constructs: (**A**) Schematic illustration of bioprinting using LAB (reproduced from Reference [81]). (**B**) Image of the bioprinting system and representative microscopic image showing the GelMA hydrogel beads array (reproduced from Reference [82]). (**C**) Generation of desmoplastic PDAC spheroids within a GleMA suspension bath (reproduced from Reference [83]). (**D**) Schematic of embedded printing-based construct and model viability after drug treatment (reproduced from Reference [84]). * *p* < 0.05, ** *p* < 0.01.

Recently, Hakobyan et al. utilized laser-assisted 3D bioprinting to generate PDAC spheroid models (Figure 4A) [81]. They bioprinted PDAC cells within layers of gelatin methacryloyl (GelMA), a gelatin derivative widely used in bioprinting applications, sequentially depositing a second layer atop the first. The construct was then crosslinked via ultraviolet light to encapsulate the bioprinted cells entirely within the hydrogel. This model demonstrated high cell viability and expression of PDAC-specific markers. Although this study did not incorporate tumor microenvironment components or drug screening assays, its approach illustrates the potential of 3D bioprinting for culturing spheroids or organoids. Similarly, our group developed a 3D bioprinting approach based on dot extrusion printing (DEP) technology to fabricate GelMA hydrogel microspheres that mimic the tumor–stroma microtissue of PDAC (Figure 4B) [82]. By precisely tuning the printing parameters, the team successfully generated uniform GelMA microspheres, which served as carriers for the co-culture of pancreatic cancer cells (BxPC-3) and stromal fibroblasts (NHDFs), forming microtissues with biomimetic ECM characteristics. Experimental results demonstrated that the microtissues could recapitulate the fibrotic microenvironment of PDAC, wherein fibroblasts were activated into a CAF-like phenotype and formed a dense stromal barrier. Drug testing revealed that these stroma-rich microtissues exhibited increased resistance to the chemotherapeutic agent gemcitabine, highlighting the critical role of the stromal barrier in mediating drug resistance. This study provides an efficient and reproducible in vitro platform for pancreatic cancer drug screening.

In order to effectively produce the PDAC microtissue on a uniform scale, we further proposed a novel embedded-dot bioprinting strategy for the one-step construction of multicellular spheroid models that recapitulate the architectural and fibrotic microenvironment of pancreatic cancer (Figure 4C) [83]. In this approach, collagen/gelatin hydrogel microspheres loaded with cells were directly deposited into a GelMA suspension bath, forming spherical multicellular aggregates (SMAs) that subsequently self-assembled into compact spheroids. This method enables precise control over spheroid size and spatial positioning, demonstrating significant potential for applications in drug screening and disease mechanism studies. More recently, Maria V. Monteiro et al. developed a method to construct a 3D tumor–stroma model of PDAC using embedded 3D bioprinting for preclinical drug screening (Figure 4D) [84]. By combining ECM-mimicking biomaterials such as GelMA and hyaluronic acid methacrylate (HAMA), they successfully fabricated PDAC models with human tumor-scale dimensions and representative stromal components. These models recapitulate the cellular and spatial stratification of the TME and maintain cell viability for up to 14 days. Experimental results demonstrated a dose-dependent response to gemcitabine treatment, with the stroma-containing co-culture models exhibiting increased drug resistance, highlighting the critical role of stromal cells in mediating chemoresistance.

### 3.4. PDAC PDOs

**Figure 5:**
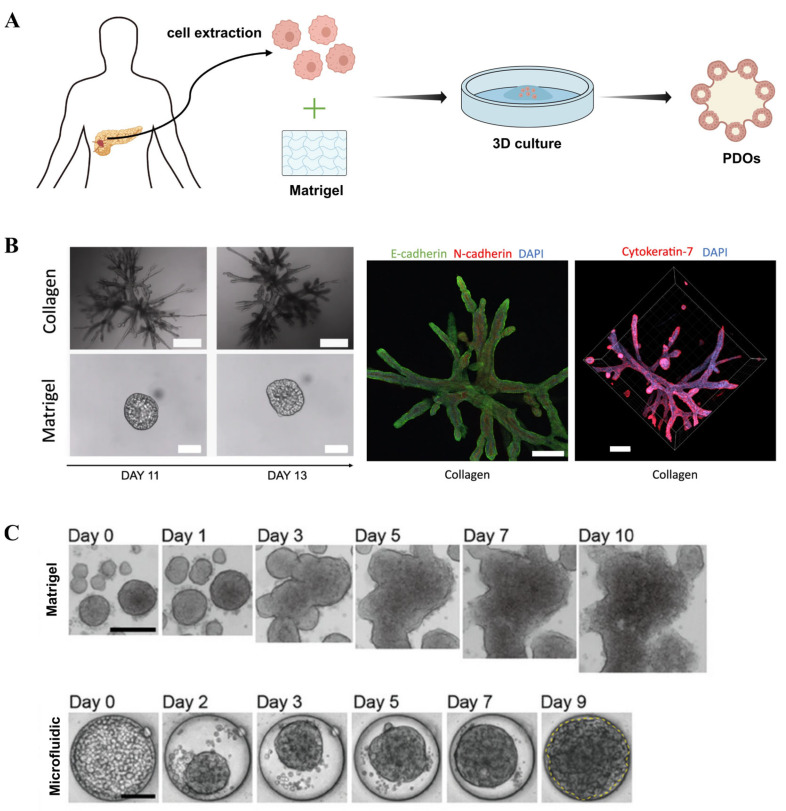
Three-dimensional tumor organoids: (**A**) Schematic illustration of PDAC tumor organoid formation. (**B**) Bright-field images showing morphological differences in organoids cultured in collagen and Matrigel (reproduced from Reference [93]). (**C**) PDAC organoids cultured using microfluidics and conventional Matrigel methods (reproduced from Reference [94]).

PDOs, which are derived from patients’ tissue, have rapidly emerged as valuable preclinical screening platforms due to their ability to recapitulate key features of in vivo solid tumors [85,86,87]. Unlike spheroids, PDOs spontaneously self-organize into 3D structures without requiring forced aggregation. Figure 5A illustrates the PDO cultivation process. More importantly, these PDOs typically exhibit a high physiological relevance by faithfully reproducing tumor-specific gene and protein expression patterns, tissue morphological characteristics, and preclinical drug responses [88]. PDOs offer unprecedented predictive accuracy, establishing them as clinically relevant in vitro tools for informing treatment decisions. Consequently, PDOs are recognized as next-generation microtumors.

Biobanks containing PDOs, CAFs, and peripheral blood lymphocytes have been established as foundational resources for engineering organoid models [89]. Recently, Susan Tsai et al. proposed a method combining PDOs with fibroblasts and incorporated lymphocytes to study T cell infiltration within the TME [90]. They co-cultured patient-derived fibroblasts with PDOs within a hydrogel matrix while allowing suspended lymphocytes to infiltrate the organoids. This group showed that co-culture with fibroblasts induces myCAF activation, which ultimately exerts a significant impact on the efficacy of gemcitabine against tumor cells. Published reports have also described the use of PDAC PDOs to evaluate the sensitivity of primary patient cells to various chemotherapeutic agents used in PDAC treatment. Additionally, these organoids have been utilized to investigate tumor responses to novel compounds [91,92].

Beyond generation of spheroidal PDOs using Matrigel dome, researchers generated PDOs with branched structures to replicate in vivo PDAC architecture by using a floating collagen gel culture system (Figure 5B) [93]. Researchers investigated the self-organization of PDAC cells into highly branched structures within a collagen matrix, elucidating four dynamic stages of branching morphogenesis: initiation, extension, thickening, and lumen formation. They proposed a minimal theoretical model based on cell proliferation, branching, and invasion to explain this process. The study also demonstrated that the properties of the ECM play a critical role in determining the branching phenotype, with collagen matrices being more conducive to the formation of complex organoid structures compared to Matrigel.

To address the issue of achieving uniform organoid size, Choi et al. developed a microfluidics-based platform for culturing PDOs, enabling personalized assessment of chemotherapy and immunotherapy efficacy (Figure 5C) [94]. By optimizing the design of the microfluidic device, the platform allowed efficient organoid cultivation from limited biopsy samples and demonstrated practical utility in drug sensitivity testing and immunotherapy evaluation. The results showed that organoids cultured in the microfluidic system exhibited comparable phenotypic and gene expression profiles to those grown in conventional Matrigel while offering greater uniformity and requiring fewer input cells.

Typically, PDOs are cultured within animal-derived biomaterials such as Matrigel or collagen. Meanwhile, the batch-to-batch variability directly impacts organoid growth efficiency, morphology, and differentiation consistency, complicating experimental reproducibility and large-scale drug screening applications. Another concern remains the limited control over biomechanical properties of Matrigel or collagen. The stiffness cannot be tuned to mimic tissue-specific ECM dynamics. While pancreatic tumors exhibit progressive ECM stiffening, a critical driver of chemoresistance that Matrigel-based models fail to recapitulate. Table 1 provides a comparative overview of 3D PDAC models, outlining their advantages and limitations.

## 4. Applications of 3D PDAC Models

### 4.1. Drug Screening

The application of 3D PDAC models has revolutionized preclinical drug screening, offering a significantly more physiologically relevant platform than traditional 2D cultures to assess therapeutic efficacy and resistance mechanisms. For example, PDAC spheroids processing tight cell–cell interactions, biomimetic 3D structures and being easily generated in a high-throughput manner, have been widely utilized for in vitro drug screening and demonstrated stronger drug resistance than the 2D cultures [95,96,97]. Bioprinted constructs introducing hydrogels as an ECM-mimetic barrier have enabled the study of PDAC-ECM dynamics in the context of drug resistance. Further, the presence of stromal components like CAFs facilitates the development of novel therapeutic strategies targeting the stromal compartment, or combination therapies, as the heterotypic interactions between tumor cells and stroma are considered a fundamental mechanism underlying chemoresistance in PDAC [98]. Previous studies have highlighted the role of the stroma and stromal-targeting approaches in both primary tumors and metastatic progression [99]. On the other hand, the use of PDAC PDOs, in particular, facilitates personalized drug testing. Generated directly from patient tumor tissue obtained via surgery or biopsy, PDOs retain the genetic, phenotypic, and functional heterogeneity of the individual patient’s tumor. This high degree of patient-specific fidelity makes PDOs exceptionally suited for functional precision oncology [100]. In practice, PDOs established from a patient’s tumor can be rapidly expanded and subjected to high-throughput drug screening against a panel of clinically relevant agents, including standard chemotherapies, targeted therapies, and novel experimental compounds [101]. While challenges remain in standardization and scalability, 3D pancreatic cancer models are increasingly recognized as indispensable tools for bridging the gap between in vitro discovery and clinical success in oncology drug development.

### 4.2. Tumor Research

3D PDAC models are ideal in vitro systems that can be used to mimic cancer oncogenesis and metastasis. Metastasis, the spread of tumor cells to distant organs, represents one of the most lethal aspects of malignant tumors [102,103,104]. It fundamentally reflects the invasive capacity of cancer cells. This complex, multi-step process is characterized by several key features: firstly, tumor cells lose their epithelial polarity and disrupt normal tissue architecture. Subsequently, they break through the basement membrane barrier. Following this breach, tumor cells intravasate, entering the bloodstream and/or lymphatic vessels. Once circulating, they extravasate out of these vessels at distant sites. The cells then migrate through the new tissue environment and finally establish themselves, expanding to form a metastatic colony. The enhanced physiological relevance makes 3D PDAC models, including spheroids, organoids, tumor-on-a-chip, and bioprinted constructs, vital for elucidating disease mechanisms and evaluating potential therapeutic strategies in a more predictive context. For instance, a human PDAC PDO model embedded in collagen hydrogels was developed to investigate the molecular mechanisms of invasion [105]. These organoids displayed two distinct invasion phenotypes, which were found to be associated with clinical features. Notably, mesenchymal-like PDOs exhibited significantly increased lethality during invasion, highlighting the potential of PDOs to reflect patient-specific invasive behavior [106]. Specifically, hydrogels with tunable stiffness constitute a suitable platform for modeling stiffness-mediated tumor invasion and stemness-related phenotypes. As reported in Liu’s work, a stiff 3D environment promoted EMT of PDAC cells and subsequently enhanced drug resistance [107].

## 5. Conclusions and Future Perspective

The desmoplastic microenvironment and unique stromal compartment of pancreatic cancer pose significant challenges for preclinical replication. Addressing the critical need for increasingly physiomimetic models in both fundamental tumor biology research and innovative therapeutic screening, we firstly reviewed and discussed the key components of the PDAC tumor microenvironment and then summarized recent advances in bioengineered and physiologically relevant 3D in vitro models, ranging from simple spheroids to complex platforms including tumor-on-chip, 3D bioprinting and tumor-derived organoids. These models offer unprecedented opportunities to recapitulate the complex architecture, cellular heterogeneity, and dynamic microenvironment of human PDAC tumors in a controlled laboratory setting, thus enabling deeper mechanistic insights into PDAC pathogenesis, stromal–tumor interactions, metastasis, and therapy resistance, thereby accelerating the discovery and validation of novel therapeutic targets.

Studies have revealed the spatial heterogeneity between CAFs and tumor cells. Spatially, CAFs in direct contact with tumor cells often form a dense physical barrier known as myCAFs, typically characterized by high expression of α-SMA. In contrast, iCAFs, which secrete pro-inflammatory cytokines such as IL-6, are usually located further away from tumor cells and interact with them primarily through paracrine signaling. This spatial heterogeneity significantly influences immuno-suppression, chemoresistance, and tumor development. Mimicking this intricate spatial organization in vitro remains a major challenge. Three-dimensional bioprinting offers a promising solution by enabling the predefined spatial arrangement of different cell types, the creation of fibrotic zones, and the modulation of hydrogel stiffness. By precisely tuning the composition and mechanical properties of hydrogels, researchers can systematically mimic the biomechanical characteristics of the pancreatic TME to investigate their specific roles in PDAC progression [108,109,110]. For example, a tunable self-assembling hydrogel system based on peptide amphiphiles was developed to model PDAC in a stiff 3D context, revealing that such mechanical conditions enhance tumor aggressiveness and drug resistance [111]. In another study, Menekse Ermis et al. cultured PDAC heterospheroids comprising cancer cells and CAFs in HAMA/GelMA hydrogels of tunable stiffness. Their results demonstrated that stiffer matrices downregulated stemness markers while upregulating EMT markers, suggesting a mechanical-driven shift toward a more invasive phenotype [9]. These findings emphasize the critical influence of biomechanical cues on PDAC behavior and provide guidance for developing personalized, biomechanically relevant pancreatic tumor models.

Furthermore, most in vitro pancreatic cancer models commonly utilize established PDAC cell lines such as Panc-1, BxPC-3, and Capan-1. These models fail to capture the individualized characteristics of different patients and cannot replicate the tumor growth state observed within the in vivo environment. Consequently, they exhibit significant limitations for applications in drug screening. PDOs, which are derived from patients, closely recapitulate the physiological structure and relevant functions of human tumor tissues in vivo, allowing the effective evaluation of drug efficacy. In recent years, research on PDAC PDOs has achieved significant progress and begun to demonstrate promise for drug testing applications. However, currently established in vitro PDAC PDO models still face substantial challenges for practical clinical translation. One of the major challenges in the construction of PDOs is the lack of standardized protocols for PDO culture and the high variability in organoid formation. Current in vitro PDO culture relies entirely on cellular self-organization, a process exhibiting considerable stochasticity. This results in low reproducibility regarding PDO size, morphology, and consistency, compromising the accuracy of drug response prediction. Another significant challenge lies in the lack of key biostructural features of the pancreatic cancer TME. Reported biomimetic organoid models of the PDAC microenvironment are typically heterotypic cultures formed by mixing multiple cell types. They often lack characteristic cellular distribution patterns, which limits their ability to faithfully recapitulate the spatial characteristics of the TME. Future research should also focus on integrating complementary technologies, for example, combining PDOs with 3D bioprinting for precise microenvironmental control. On the other hand, integrating functional vasculature or microfluidic systems is essential for modeling nutrient/oxygen gradients, drug delivery, and metastasis.

Ultimately, the continuous evolution of these bioengineered 3D PDAC models promises to bridge the gap between traditional preclinical models and human patients. By offering more predictive platforms for drug testing and fostering a deeper understanding of PDAC biology, these advanced in vitro systems hold immense potential to revolutionize therapeutic development and propel us towards more effective treatments for this disease.

## Figures and Tables

**Table 1:** Advantages and limitations of in vitro 3D PDAC models.

3D PDAC Model	Characteristics	Advantages	Limitations
3D spheroids	Aggregate of multiple cells, driven by cell–cell attachment and ECM secretion	Easy production, high-throughput allows co-cultures	Not all cell lines form spheroids, necrotic cores Lack vascularized
Tumor-on-a-chip	Cells cultured with a designed chip	Allow co-cultures Vascularized Dynamic culture	Costly Requires special equipment Difficult to scale up
3D bioprinting	Controlled layer-by-layer deposition of cells following a computer-programmed design	Precise position of multiple cells High-throughput In vivo-like complexity	Costly Require special equipment Challenges with cells/materials
PDAC PDOs	Cultured in a hydrogel environment	Formed from primary cells Patient-specific In vivo-like complexity/architecture	Establishment can take a long time No standardized protocol Limited in size

## Data Availability

Not applicable.
